# Genomic insights into bacterial kidney disease resistance in Arctic charr (*Salvelinus alpinus*) using a newly developed 71k SNP array

**DOI:** 10.1186/s12711-026-01118-w

**Published:** 2026-11-14

**Authors:** Christos Palaiokostas, Henrik Jeuthe, Katja Nilsson, Hampus Hällbom, Charlotte Axén, Øystein Evensen, Susanne Eriksson, Martin Johnsson

**Affiliations:** 1 Department of Animal Biosciences, Swedish University of Agricultural Sciences, Uppsala, Sweden; 2 Aquaculture Center North, Åvägen 17, Kälarne, 844 61 Sweden; 3 Swedish Veterinary Agency, Ulls väg 2B, Uppsala, 751 89 Sweden; 4 Department of Paraclinical Sciences, Faculty of Veterinary Medicine, Norwegian University of Life Sciences, Ås, Norway

## Abstract

**Background:**

Selection for disease resistance forms one of the most highlighted areas of aquaculture breeding. A breeding program for Arctic charr has been operating in Sweden for over 40 years, making it the oldest of its kind worldwide for this species. However, the lack of available genomic resources has so far prevented selection for any disease-resistance traits. A 71k Axiom SNP array was produced in this study and used to assess the potential to select for charr resistant to bacterial kidney disease (BKD), which is currently a major threat to the industry.

**Results:**

Following a challenge experiment with *Renibacterium salmoninarum*, the causative agent of BKD, we collected relevant phenotypic proxies from approximately 2000 charr juveniles. We then genotyped these animals with the new 71k SNP array. The magnitude of the estimated variance components suggested a potential for BKD resistance breeding in charr, with heritabilities ranging from 0.05 to 0.56 depending on the resistance proxy used. The conducted genome-wide association studies (GWAS) suggested that BKD resistance is a polygenic trait. Furthermore, genomic prediction approaches indicated that BKD-resistant animals can be identified using their SNP genotypes. Accuracies, expressed as Pearson correlation coefficients, when BKD resistance was analysed as a continuous trait, ranged from 0.42 to 0.52. On the other hand, when BKD resistance was treated as a binary trait, the efficiency of genomic prediction was assessed using ROC curves, with an area under the curve equal to 0.72. Finally, a preliminary analysis did not point towards any unfavourable correlations with growth traits.

**Conclusions:**

The developed 71k SNP array has the potential of being a pivotal tool for the Swedish Arctic charr breeding program. Moreover, our data support the use of genomic prediction in breeding BKD-resistant Arctic charr. As a critical next step, further validation in actual industry conditions is required.

**Supplementary Information:**

The online version contains supplementary material available at https://doi.org/10.1186/s12711-026-01118-w.

## Background

Bacterial kidney disease (BKD), is caused by *Renibacterium salmoninarum*, a gram positive, facultative intracellular, rod-shaped bacterium. The bacterium is most active at temperatures under 15 °C [1] and it is transmitted both vertically and horizontally [2]. The disease poses a significant threat to salmonid farming [3], among which Arctic charr (*Salvelinus alpinus*) is particularly vulnerable. In Sweden, BKD is considered the most damaging disease for the domestic charr industry, with recorded mortalities reaching up to 80% [4]. The lack of effective therapeutic agents exacerbates the problem, and the most effective way to control the infection is to slaughter the animals and disinfect the facility [5]. Therefore, the disease is listed and subject to management measures according to national legislation (SJVFS 2024:6/K134). Breeding for genetically resistant animals might offer solutions and mitigate the problem [6].

Considerable effort has been devoted to selecting for disease resistance across various aquaculture species [7]. This is hardly surprising, as the most common production system in aquaculture, which uses net pens in lakes or the sea, entails constant interaction with the surrounding aquatic environment. As such, any treatment directly affects the ecosystem, limiting the pharmaceuticals available. Furthermore, as aquaculture is a relatively recent industry, effective therapeutic agents remain scarce for many diseases [8].

The adoption of genomics in aquaculture breeding has propelled progress, enabling the detection of genes associated with genetic resistance [9–12] and the implementation of genomic selection [13] in commercial breeding programs [14–16]. As is typical with disease-resistance traits, the breeding candidate itself does not have a phenotype, and selection relies on information from relatives. Including genomic information in such cases has repeatedly been shown to substantially improve selection accuracy compared with traditional pedigree-based approaches [17–23].

Similar to livestock, single-nucleotide polymorphism (SNP) arrays are the tool of choice for modern breeding practices in aquaculture species. Although genotype-by-sequencing approaches have been shown to be valuable [24], SNP array genotypes are generally considered more reliable because several quality control steps are implemented during construction. Furthermore, their simpler downstream analysis requirements largely explain their widespread adoption in breeding.

A national breeding program for Arctic charr has been operating in Sweden for over 40 years, focusing primarily on growth-related traits [25]. The program has not previously included any trait related to disease resistance in its breeding goals. To the best of our knowledge, the potential to select for BKD-resistant charr is unknown, as no prior heritability estimates are available. Notably, a moderate heritability of approximately 0.23 has been reported for Atlantic salmon (*Salmo salar*) [26].

Until recently, the limited availability of genomic resources has restricted the implementation of genomic-based breeding decisions. As such, the Arctic charr program has been relying entirely on pedigree-derived breeding values. The recent development of a high-density Axiom array (Thermo Fisher Scientific™) containing approximately 600,000 SNPs [27] has been pivotal for the future of the breeding program, enabling genomic selection.

Globally, Arctic charr is a niche aquaculture species, and although it is the second most farmed fish in Sweden, its annual production does not exceed 1500 tonnes. Thus, the routine use of a high-density array is financially challenging. Previous studies in various aquaculture species have shown that genomic selection can be successfully implemented with relatively low SNP densities [28–31]. Since SNP density is a key driver of genotyping costs, lower densities would reduce per-sample costs without compromising selection efficiency [32]. Naturally, the above also depends on the actual underlying genetic architecture [33]. Regardless, linkage disequilibrium profiles previously obtained from genotyping charr from several year classes of the breeding programs using the high-density array suggested that a low- to medium-density array would be suitable for genomic selection [27].

In the current study, we developed a medium-density array containing 71k SNPs selected from the aforementioned high-density one. We phenotyped approximately 2000 Arctic charr juveniles from the 2025 year-class of the Swedish breeding programme for proxies associated with BKD resistance. These phenotypes, derived from a disease challenge experiment, included survival (as a binary trait), number of surviving days, and bacterial load (estimated by RT-qPCR). More specifically, overall survival is the most commonly recorded trait in disease challenge experiments, as it reflects an animal’s resilience to a pathogen and its ability to survive a particular infection. However, reducing such a complex trait as disease resistance to a binary trait (e.g., overall survival) usually does not suffice [34]. Therefore, we included the number of surviving days and the measured bacterial load in the analysis. The former has been associated with an animal’s susceptibility [35], while the latter is a more direct metric, potentially allowing the disentanglement of a host’s ability to nullify the pathogen from its ability to tolerate high loads of it [36].

All the animals from the disease challenge experiment were genotyped with the newly developed 71k SNP array. Heritability estimates for the resistance proxies were estimated, followed by genome-wide association analysis (GWAS). Finally, we investigated the potential of using genomic selection to identify genetically resistant animals.

## Methods

### Medium-density array development

​The selection of SNPs for the high-density Arctic charr (designed based on information from Nordic charr populations) array has been previously described [27]. Subsequent filtering of those markers for ​developing a medium-density array used information from only the Swedish selectively bred population and involved a minimum call rate of 99%, a minimum minor allele frequency of 0.15, and no statistically significant deviations from Hardy-Weinberg equilibrium (α = 0.001). Moreover, we selected only SNPs with a minimum physical distance of 5 kbp between them. Following further recommendations from Thermo Fisher Scientific, 71k (71,301 tiled SNPs) of the above SNPs were used to construct an Axiom array, trademarked as *Salpinus*. Those recommendations were derived from their proprietary software, which provides a probabilistic estimate of the likelihood that a given SNP will convert into a reliable SNP assay.

### Background of the studied population

Our study used Arctic charr from the Swedish national breeding programme [37]. The base population originates from wild fish collected in 1979 in Lake Hornavan, northern Sweden. The initial number of founding animals is unknown, with recent estimates suggesting an effective population size (N_e_) of approximately 20 [27]. The programme operates within a closed breeding nucleus, with discrete generations, using a 1:2 sire-to-dam mating ratio. The number of families in each generation has ranged from 45 to 125. Currently, the 10th generation of the breeding programme has been formed, comprising 89 full-sib families.

### BKD challenge experiment and phenotypic recordings

The bacterial strain used for the disease challenge experiment was the SVA9 (4/86). It originated from an earlier Swedish BKD outbreak and has been utilised in previous studies [38]. The bacterium was initially cultivated on KDMC agar plates. Colonies were harvested and transferred to KDM broth, then incubated on a shaking platform at 15 °C. The OD was monitored with a PV4 visible spectrophotometer until the broth reached an estimated bacterial concentration of 5 × 10^7^ CFU/ml.

Juveniles from the 10th generation of the Swedish Arctic charr breeding program were transferred to VESO Aqualab in Norway during June 2025. In total, 2200 fish (2–5 g) from 89 full-sib families were used. The animals were acclimated over a growth period of approximately two months, during which they reached an average weight of ~ 20 g. Thereafter, 2000 of those animals were infected through intraperitoneal (IP) injection with the prepared bacterium inoculum (0.1 ml per animal). The injected animals were split equally between two tanks (each containing 1000 animals) and monitored daily in accordance with VESO Aqualab’s standard operating procedures. This involved continuous monitoring of water quality parameters, collection of dead fish, and observation of abnormal behaviour in the challenged animals (e.g., reduced swimming capacity, loss of appetite). The freshwater temperature was 12 °C (± 1 °C), with a water flow of 0.8 L/kg/min, while a photoperiod of 24:0 (L: D) was used throughout the study.

During the experiment, fin clips were collected from all fish for genotyping. Furthermore, head kidney tissue was collected from 800 animals for RT-qPCR analysis to assess bacterial load. Those animals included approximately 400 that were among the first mortalities, while the rest survived until near the end of the experiment. The RT-qPCR analysis was performed at PatoGen’s facilities in Ålesund, Norway, using their accredited protocols (Norwegian Standard NS-EN ISO/IEC 17025). Ct values, representing the cycle number at which fluorescence surpasses the detection threshold, were produced for each sample as indicators of bacterial load. In general, the higher the initial bacterial load in a sample, the fewer PCR cycles are needed to produce a detectable amount of PCR product.

Overall, the following BKD resistance proxies were used for downstream analysis: days of survival (continuous trait), overall survival, and N_50_ survival (binary traits). The latter trait indicated whether an animal was alive when the population reached 50% mortality. Finally, for the subset of animals from RT-qPCR, the Ct values (a continuous trait) were analysed as a proxy for BKD resistance.

### Genoty​ping and quality control

Genotyping was performed on all 2000 charr juveniles challenged with *Renibacterium salmoninarum*. Genomic DNA was extracted from collected fin-clips, followed by genotyping with the new custom Axiom™ SNP array. Both activities were performed by IdentiGEN (Dublin, Ireland). The obtained genotypic data were filtered using preGSf90 v1.26 from the BLUPf90 suite [39], discarding SNPs with a call rate below 98% and minor allele frequency (MAF) below 0.05. Moreover, the same software was used to filter SNPs for which the number of observed heterozygotes deviated from the number expected under the Hardy-Weinberg equilibrium by more than 0.15 [40]. Finally, the existence of any underlying population structure was assessed using principal component analysis (PCA) on the genomic relationship matrix (GRM). The GRM was estimated with preGSf90 v1.26 from the BLUPf90 software suite, following VanRaden (2008) [41].

### Parentage assignment and pedigree reconstruction

During transport, all the animals were pooled in the same tank container. Because the fish were not tagged, each individual’s pedigree was unknown. A subset of candidate parents had been previously genotyped (*n* = 96) with the high-density array [27]. Those genotypes were subsetted for the SNPs of the current array. Parentage assignment was performed using the seekparentf90 v1.59 from the BLUPF90 suite [39], allowing for a genotypic error of 2%. For verification purposes, parentage assignment was also performed using the R package APIS v2.0.8 [42, 43]. In parallel, a naive approach to reconstructing the pedigree was performed using the GRM estimated with preGSf90 v1.26 from the BLUPf90 software suite, following VanRaden (2008) [41]. More specifically, only the off-diagonal elements of the GRM whose normalised values (each value was divided by the square root of the product of the corresponding diagonal elements; this was performed to obtain realised relationships) fell within the range 0.45–0.65 were retained as indicative of potential full-sibs. Thereafter, a network analysis was conducted using igraph v2.2.1 [44], and the resulting clusters indicated full-sib families (https://github.com/chpalaiokostas/Genetic_resistance_BKD/tree/main/Pedigree_reconstruction).

### Estimation of variance components

As phenotypic proxies of BKD resistance, we used the number of surviving days both as a continuous and a right-censored trait, overall survival, N_50_ survival and the Ct values from RT-qPCR. Variance components for those resistance proxies were estimated with blupf90 + v2.73 and gibbsf90 + v3.24 from the BLUPf90 suite [39] using the following animal model:

1 $${\bf{y}} = {\bf{Xb}} + {\bf{Zu}} + {\bf{e}},$$

where **b** is the vector of the fixed effects (intercept, tank); **X** is the incidence matrix relating phenotypes with the fixed effects; **Z** is the incidence matrix relating phenotypes with the random animal effects; **u** is the vector of random animal effects ~ N(0, **G**σ_g_^2^), where **G** is the GRM [41], σ_g_^2^ is the additive genetic variance; **e** the vector of residuals ~ N(0, **I**) and σ_e_^2^ is the residual variance. The heritability was estimated using the following formula:

$$\:{h}^{2}=\:\frac{{\sigma}_{g}^{2}}{{{\sigma}_{g}^{2}+\sigma}_{e}^{2}}$$

For the overall and N_50_ survival, the probit link function was used to map the observed binary phenotype (0 = Non-resistant, 1 = Resistant) onto the underlying liability scale. The residual variance on the underlying scale is not identifiable in threshold models [45] and was therefore fixed to 1. The parameters of each of the above models were estimated through Markov chain Monte Carlo (MCMC) using Gibbs sampling. Each MCMC involved 1 M iterations, out of which the first 100,000 were used as burn-in and a thinning step of 1000.

Finally, we tested the efficiency of a No-U-turn sampler [46] as an alternative to Gibbs sampling for estimating variance components for the binary proxies of BKD resistance. The software for Bayesian data analysis Stan [47] was used through the R package brms v2.23 [48]. We used 4 chains of 10,000 iterations each with a warm-up phase of 5000 iterations (https://github.com/chpalaiokostas/Genetic_resistance_BKD/tree/main/Bayesian_heritability_estimation).

### Genome-wide association analysis

A weighted genomic best linear unbiased predict​ion (WGBLUP) analysis was performed [49, 50] using the BLUPF90 software suite, as previously described [48], separately for surviving days, N_50_ survival and Ct values. In short, a genomic relationship matrix (GRM) was estimated following VanRaden (2008) as:

$$G =\:\frac{ZD{Z}^{{\prime\:}}}{2\:\sum\:{p}_{i}(1-{p}_{i})}$$

where **Z** *is* a matrix of centered genotypes, **D** is a weight matrix for all SNPs, and p_i_ is the corresponding MAF of each SNP. Thereafter, SNP weights were calculated using the nonlinear A method [41]. The above process was followed until convergence of the SNP weights as previously described [51]. Briefly, convergence was assessed using the following criterion.

$$\:C =\:\frac{\sum_{i}{({w}_{i}-\:{w}_{i-1})}^{2}}{{\sum_{i}w}_{i}^{2}},$$

where w_i_ denotes a SNP weight and i is the iteration number. Convergence was assumed when C was below 10^− 8^.

P-values of the estimated SNP effects were obtained with postGSf90 [52]. Statistical significance was assessed based on the Bonferroni criterion: 0.05/N, where N represents the number of SNPs retained after QC. Finally, the percentage of the explained additive genetic variance was estimated using non-overlapping windows of adjacent SNPs, each covering a physical distance of 1 Mbp.

### Genomic prediction for BKD resistance

An assessment of the efficiency of predicting BKD resistance in charr based on their SNP genotypes was conducted. Different approaches were used depending on whether the resistance proxy was the number of days of survival or a binary outcome. The dataset was split into training and validation/test sets. The size of the splits varied depending on the BKD resistance proxies used, as described below. Overall, SNP effects were estimated from direct genomic values (DGVs) derived from GBLUP [49]. Thereafter, those SNP effects were used to estimate DGVs for animals in the test/validation se​ts where the phenotypes had previously been masked.

For the surviving days, a three-fold cross-validation approach was used. More specifically, the dataset was split into sequential training (*n* = 1337) and validation sets (*n* = 667). In the validation sets, the animals’ phenotypes were masked, and their DGVs were estimated using the SNP effects derived from the training set. This cross-validation procedure was repeated 5 times to minimize potential bias arising from the random allocation of animals to the training and validation sets. The blupf90 + v2.73 and the postGSf90 v1.89 were used to estimate GEBVs and SNP effects, respectively, in the training sets. DGV predictions for the test sets were performed using predf90 v2.04 from the BLUPf90 suite [39]. The accuracy of the DGVs from the validation set was assessed using the Pearson correlation coefficient between them and the number of surviving days. Furthermore, the adjusted for fixed-effects phenotypes of the test set were regressed on the DGVs to assess potential bias. The regression slope indicates inflated values when above one and deflated values when below one. By contrast, a value equal to 1 suggests that the results are unbiased. The same approach was also used for Ct values though in this case the training and validation groups consisted of 532 and 268 animals, respectively.

In the case where BKD resistance was studied as a binary trait (N_50_ survival), SNP effects were estimated from DGVs derived from animals of a single tank (*n* = 1000). Therefore, half of the dataset (animals from either of the two tanks) was used for training, with the other half forming the test set. This approach was repeated for both tanks. Since the animals were randomly allocated to the two tanks during the challenge experiment, no biases due to unequal family representation were expected. The gibbsf90 + v3.24 and the postGSf90 v1.89 were used to estimate GEBVs and SNP effects, respectively, in the training sets. In the case of Gibbs sampling, the MCMC parameters involved 1 M iterations, of which the first 100,000 were used as burn-in. In addition, a 1000-iteration thinning step was included. As in surviving days, the predf90 v2.04 from the BLUPf90 suite [39] was used to estimate GEBVs for the animals in the test set. The assessment in this case was initially performed using a naive approach, in which animals from the test set with a DGV > 0 were classified as resistant. Equally, animals with a negative DGV were classified as non-resistant. A confusion matrix was computed to assess the model’s classifications against the actual phenotype. At the same time, receiver operator characteristic (ROC) curves were also used to assess the efficacy of classifying charr as resistant or non-resistant (based on N_50_ survival). The area under the curve (AUC) [53, 54] was used to assess performance, with a value of 1 indicating a perfect classifier.

### Associations between growth and BKD EBVs

A preliminary analysis was conducted to investigate associations between EBVs for growth and BKD. Breeding values for BKD resistance (surviving days) were estimated for the identified parents of the challenged charr. Simultaneously, growth-related EBVs were available for those animals, as they were routinely estimated for the Arctic charr breeding program. Those EBVs were estimated using the blupf90 + v2.73 [39]. BKD resistance EBVs from 36 broodfish were regressed against each animal’s corresponding growth EBV. In addition, a Pearson correlation coefficient was estimated between growth and BKD resistance EBVs.

## Results

### Descriptive analysis of BKD resistance traits

**Fig. 1:**
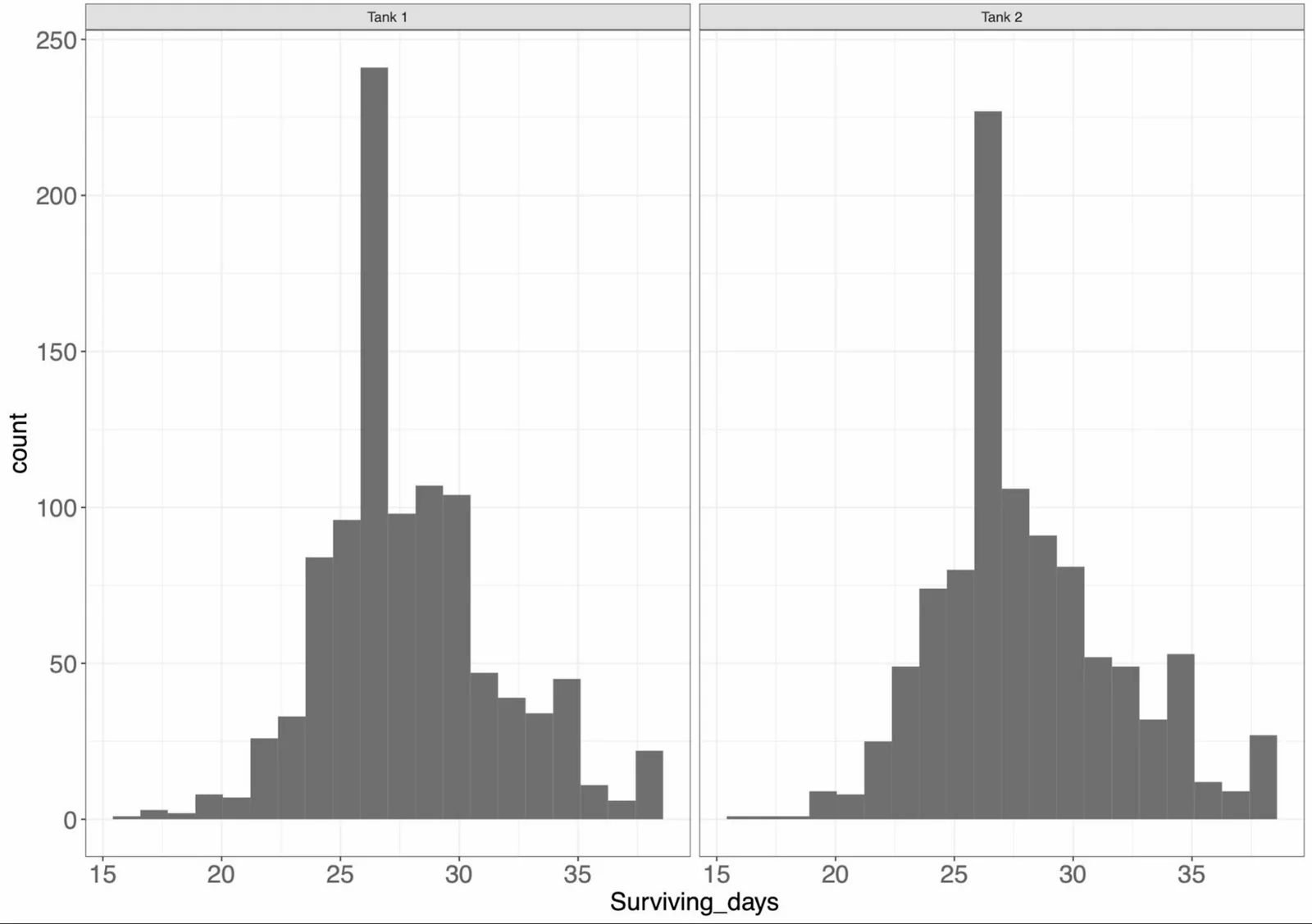
Surviving days of Arctic charr juveniles post-challenge intraperitoneal injection (IP) with an inoculum containing *Renibacterium salmoninarum* (5 × 10^6^ CFU/fish). Following infection, the fish were randomly assigned to two tanks

The challenge trial was concluded 38 days after infection. Mortalities began 16 days post-challenge and peaked during the fourth week (Fig. 1). Overall, only ~ 2% of the challenged population survived with no evident differences between the two tanks.

**Fig. 2:**
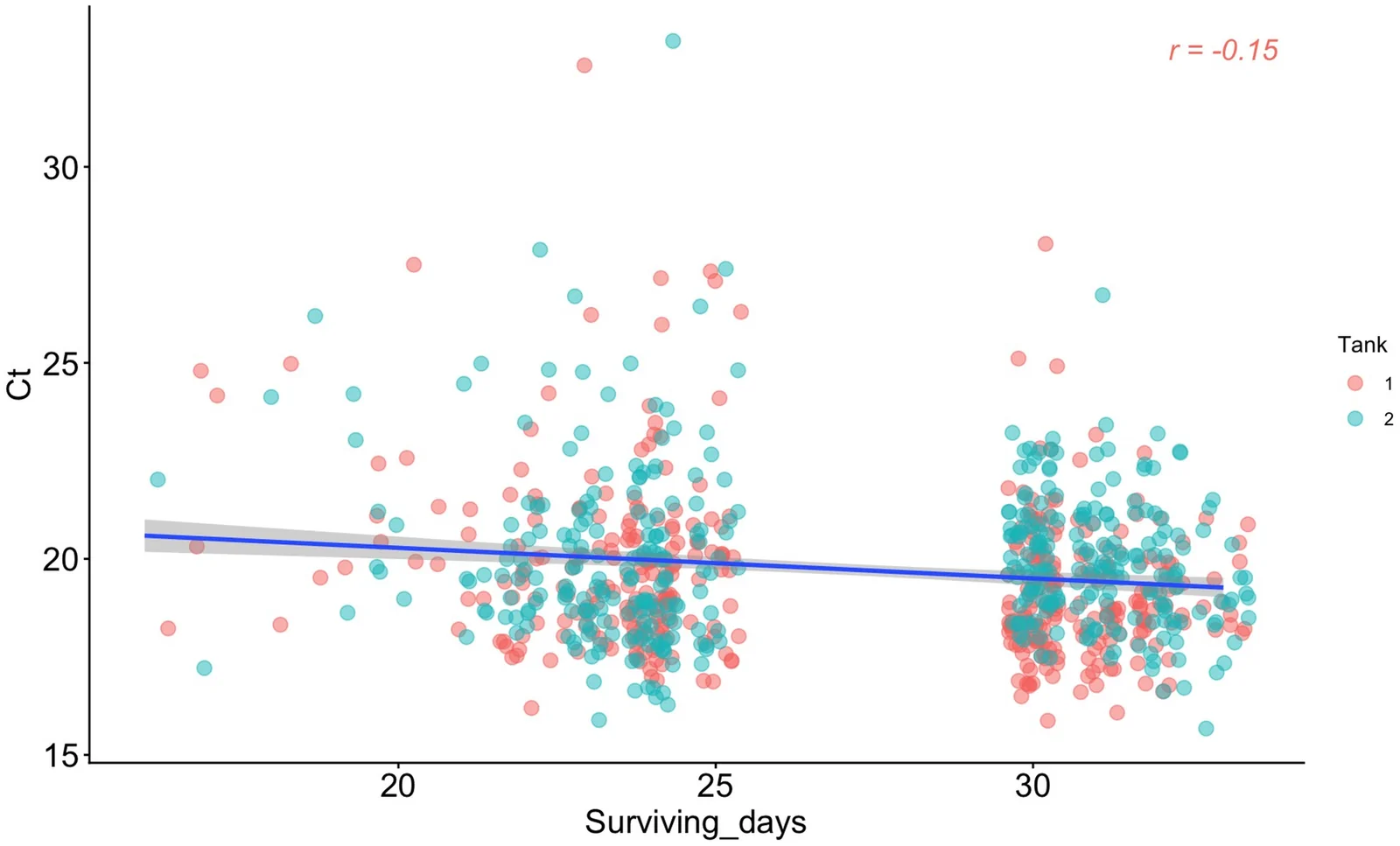
Scatterplot of Ct values obtained through RT-qPCR versus surviving days from Arctic charr juveniles challenged with *Renibacterium salmoninarum*. The plot depicts a regression line in blue, with 95% intervals in grey

A subset of the challenged animals, which either died early or survived towards the end of the experiment (*n* = 800), were sampled for RT-qPCR. No differences were observed in the obtained Ct values among the animals in the two tanks (Fig. 2). The Ct values had a negative and statistically significant association with surviving days. A regression coefficient of -0.08 was estimated (SE 0.02; *P* < 10^− 5^), while the Pearson correlation coefficient between Ct values and surviving days was − 0.15 (*P* < 1-e04; 95% -0.22 to -0.08).

### Quality control of SNP genotypes

**Fig. 3:**
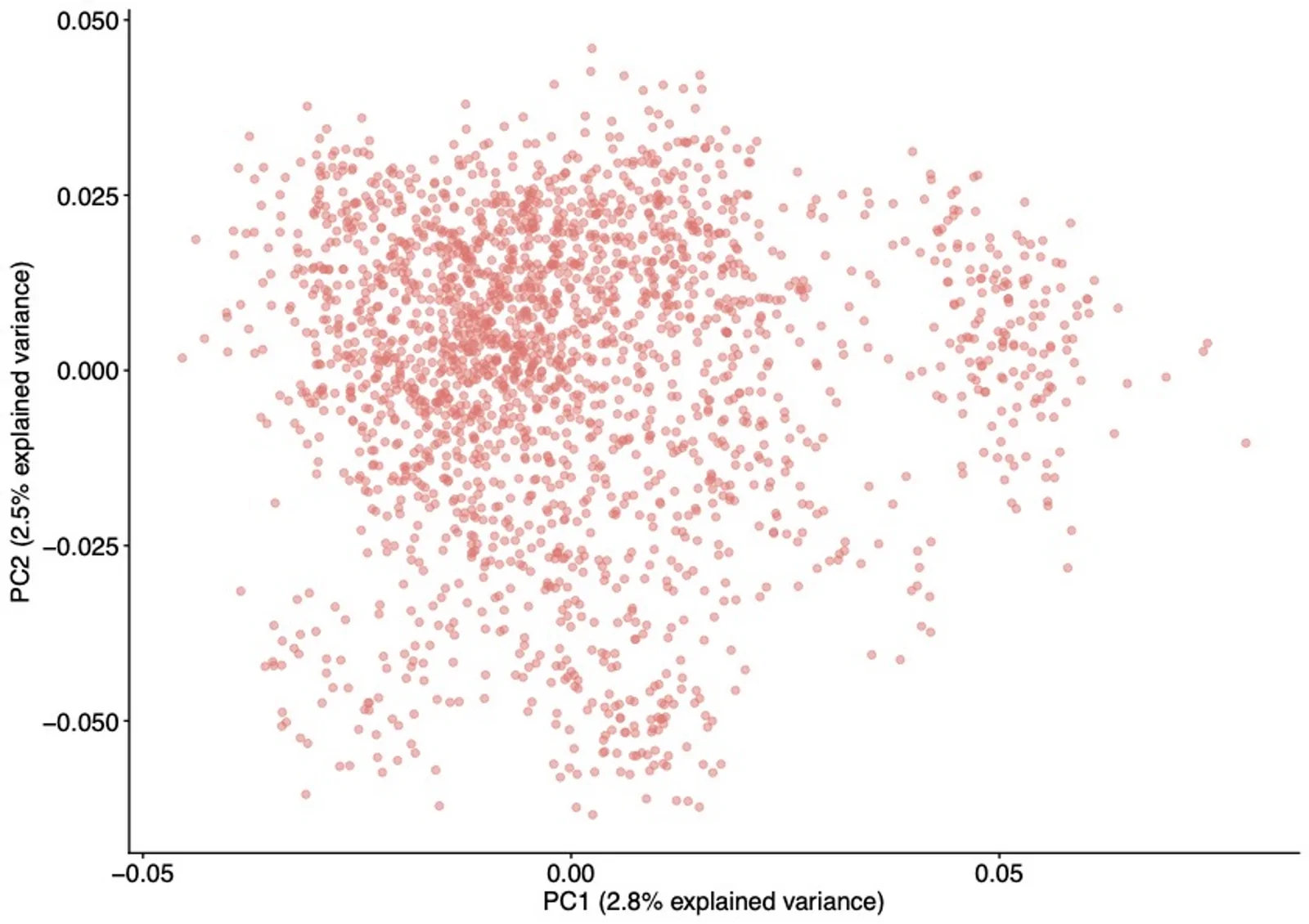
Principal component analysis of BKD-challenged Arctic charr juveniles (*n* = 1982). The PCA was performed on the genomic relationship matrix computed from approximately 70,000 SNPs. The first two principal components accounted for 2.8% and 2.5% of the observed variation, respectively

During QC, 18 animals were removed due to a call-rate below 90%. Moreover, 797 variants were removed due to MAF (< 0.05), 908 variants due to call-rate below 98%, and 31 variants were removed due to deviations from the Hardy-Weinberg equilibrium. Overall, 1982 animals and 69,745 SNPs were retained for downstream analysis. The PCA, as expected, did not reveal any obvious population structure or family clustering, with the first two principal components accounting for 2.8% and 2.5% of the observed variance, respectively (Fig. 3).

### Parentage assignment and pedigree reconstruction

**Fig. 4:**
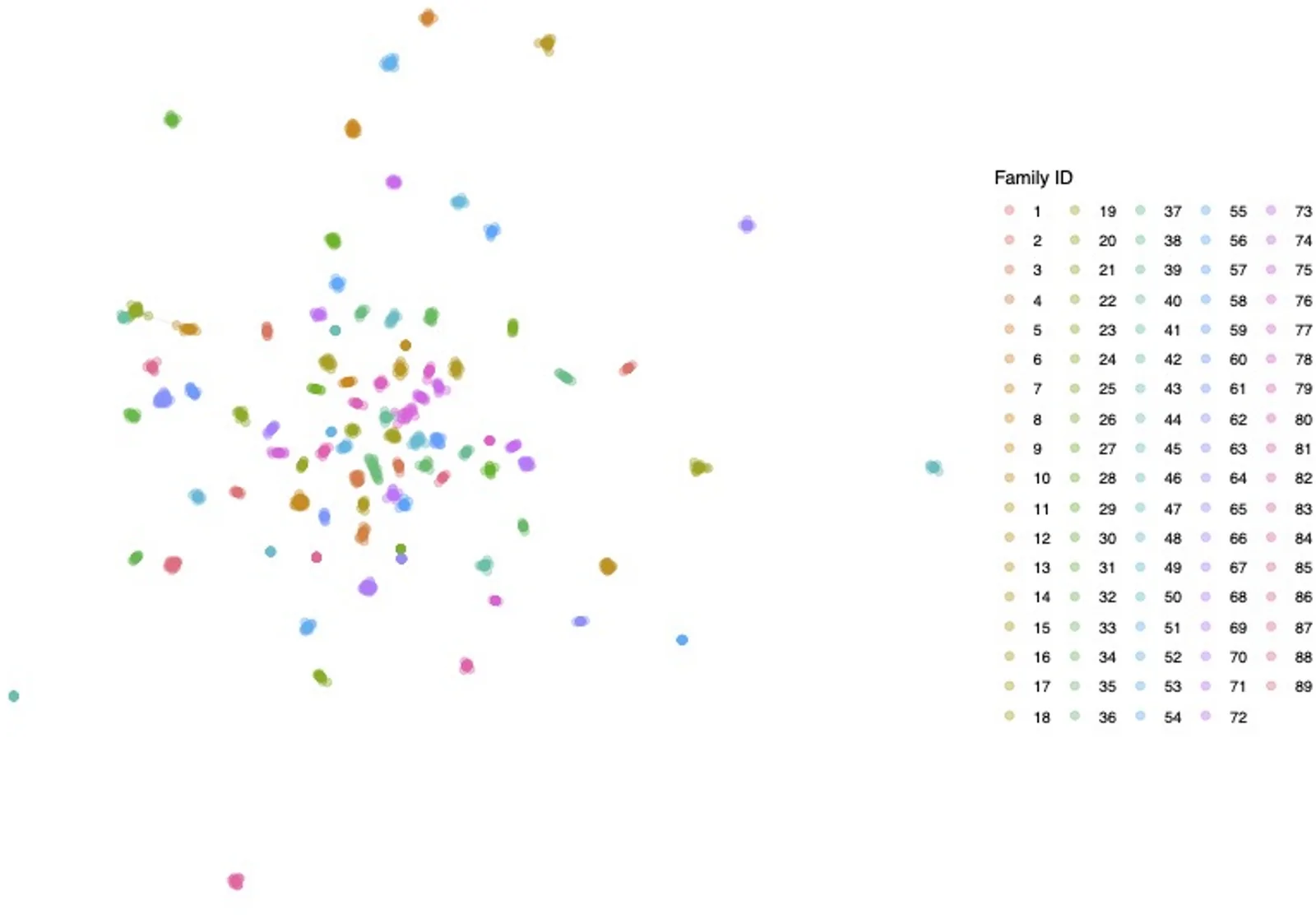
Pedigree reconstruction. Normalized genomic relationship values in the range of 0.45–0.65 were used for a network analysis conducted using the R package igraph v2.2.1. The suggested clusters indicate separate full-sib families

Allowing for a maximum genotyping error of 2% in seekparentf90 resulted in 450 charr being assigned to unique parental pairs. These assignments were in full agreement with the ones from R/APIS. The animals uniquely assigned to a parental pair represented 18 full-sib families, each with 13 to 41 offspring. The mean size of those full-sib families was 24 offspring. In addition, 1454 were assigned to at least a unique sire or dam. In the case of sires, 1032 charr were assigned, which represented 30 paternal half-sib families, each with 11 to 71 offspring. The mean offspring size of those half-sib families was 34. In the case of dams, 831 were assigned, representing 36 families, each with 13 to 41 offspring. Since each dam was mated with only one sire, those families were actually full-sib ones. The mean number of offspring in those families was 22. The network analysis conducted using the relationship values from the GRM (normalized values between 0.45 and 0.65) suggested the existence of 89 clusters (Fig. 4; Additional file 2, Figure S1), in agreement with the a priori expectation of the full-sib families used in the BKD challenge experiment.

### Estimation of variance components

The estimated heritabilities ranged from low to moderate, depending on the proxy used to characterise resistance to BKD (Table 1; Additional file 1, Tables S1 and S2). In the case of Ct (related to bacterial load), where only a subset of animals were phenotyped (*n* = 800), a low heritability estimate of 0.054 (SE 0.03) was found. Treating the number of surviving days as a proxy for resistance suggested a heritability of 0.46 (SE = 0.04). However, when the number of surviving days was treated as a right-censored trait the heritability dropped to 0.16 (SE = 0.03). On the other hand, when resistance was treated as a binary trait, heritabilities of 0.27 and 0.56 on the underlying scale were obtained for overall and N_50_ survival, respectively. Notably, especially in the former, the estimate was particularly imprecise, as the standard error exceeded half the estimate (Table 1; Additional file 1, Table S2).

**Table 1:** Heritability estimates and accompanying standard errors for proxies related to BKD resistance

Trait	Trait type	No. phenotypes	Software	Heritability	Standard Error
Surviving days	Continuous	1982	blupf90+	0.46	0.04
Surviving days censored	Right censored	1982	gibbsf90+	0.16	0.03
Overall survival	Binary	1982	gibbsf90+	0.27	0.15
N_50_ survival	Binary	1982	gibbsf90+	0.56	0.15
Ct (RT-qPCR)	Continuous	800	blupf90+	0.054	0.038

The heritabilities for the binary traits are presented on the liability scale

Most likely, this was because one class (non-resistant) was overrepresented, as only 2% of the population survived the challenge experiment. In this case, the MCMC convergence appeared suboptimal, with an estimated autocorrelation coefficient of 0.5. The analysis based on NUTS instead of Gibbs sampling resulted in mean heritability estimates of 0.58 and 0.61 for overall and N_50_ survival, respectively. The 95% highest posterior densities in this case were for those two traits: 0.08–0.94 and 0.53–0.70.

### GWAS for surviving days and N50 survival

**Fig. 5:**
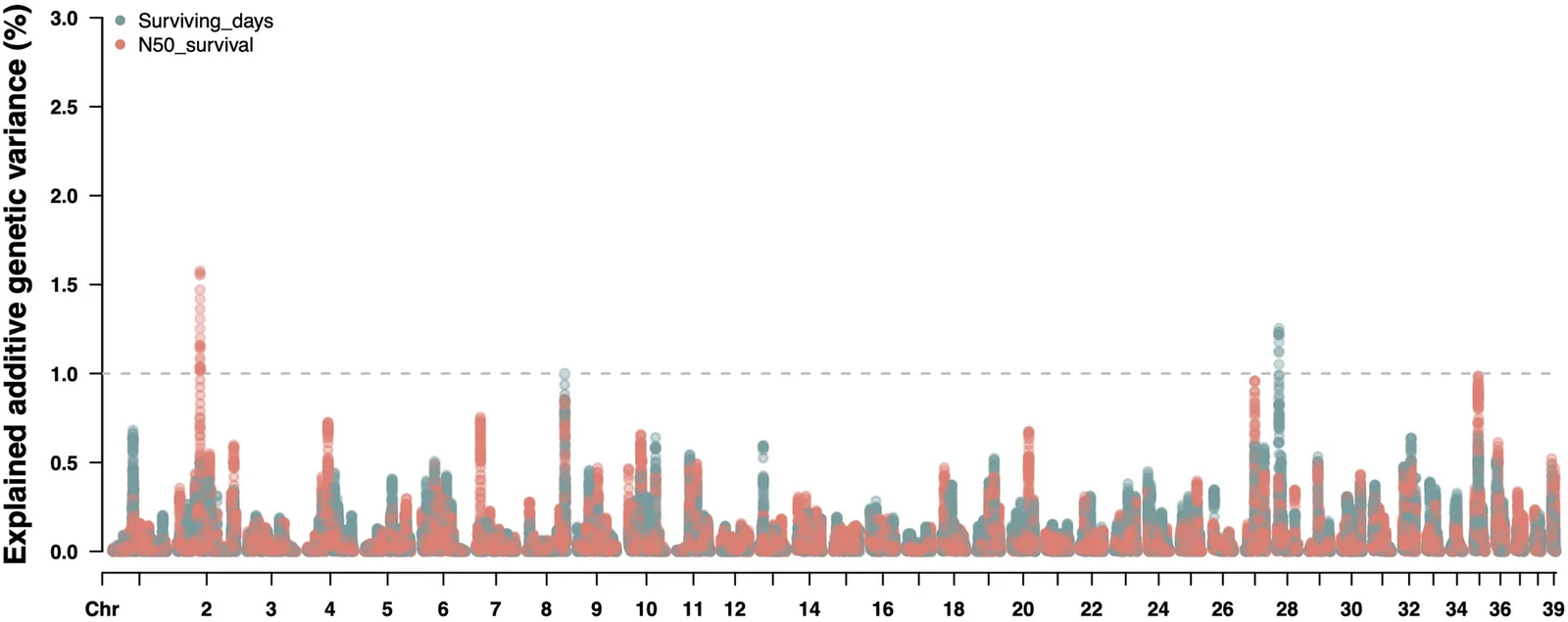
Manhattan plot showing the explained additive genetic variance for BKD resistance across the Arctic charr genome. The calculations were performed using WGBLUP with non-overlapping 1 Mbp windows. Two proxies of BKD resistance are depicted: Surviving days and N_50_ survival, each coded in a different colour

The GWAS results suggested that genetic resistance to BKD is a polygenic trait (Fig. 5; Additional file 2, Figure S2). Regions explaining just over 1% of the additive genetic variance were found in chromosomes 2, 8, 27, 28, and 35. A region on chromosome 2 accounted for the largest proportion of additive genetic variance, approximately 1.6%.

### GWAS for Ct values

**Fig. 6:**
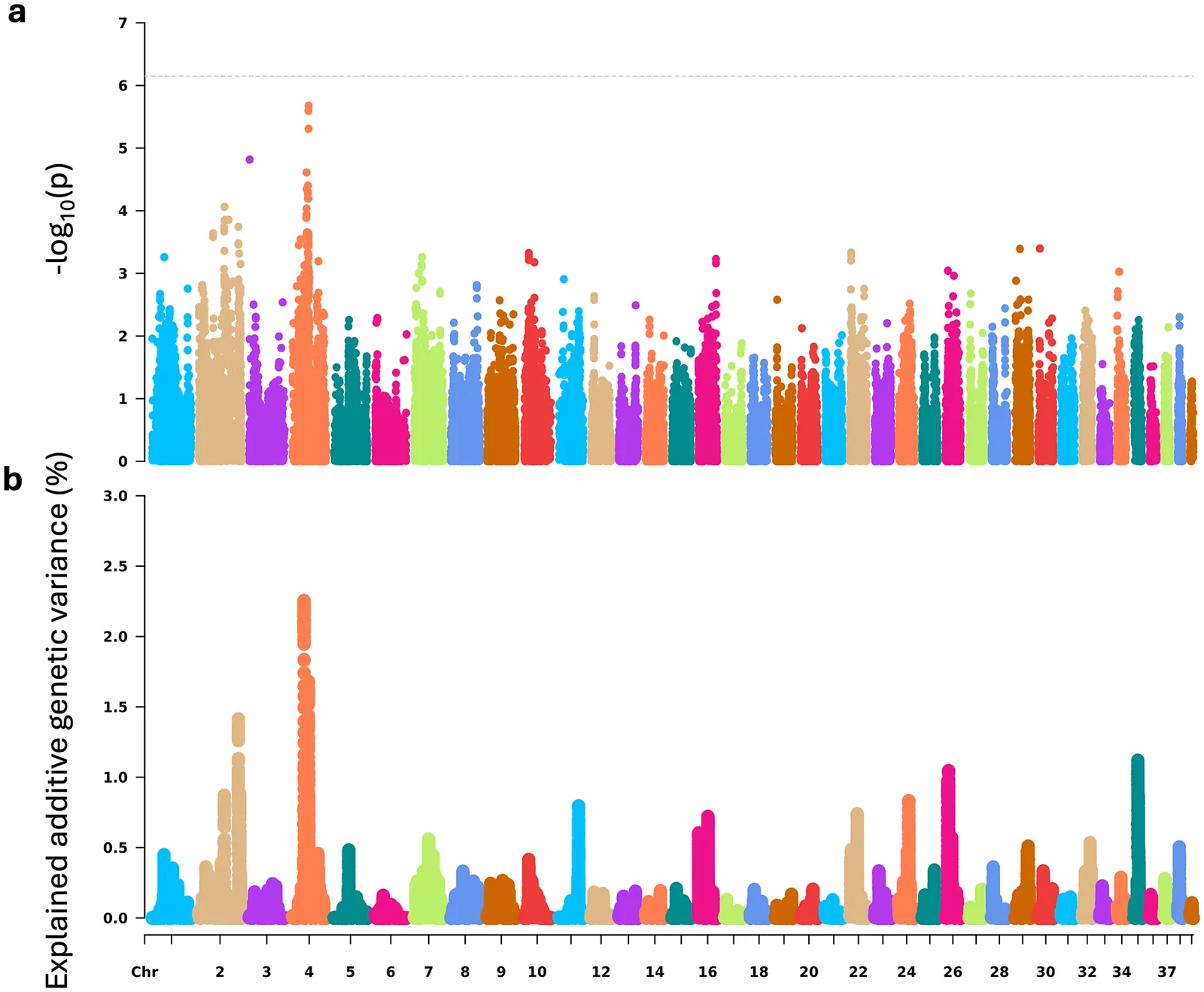
Manhattan plots for Ct and associated additive genetic variance. (**a**) Manhattan plot depicting -log10(p-values) estimated from WGBLUP for Ct values across the Arctic charr genome. (**b**) Manhattan plot depicting the explained additive genetic variance for Ct values across the Arctic charr genome. The calculations were performed using WGBLUP with non-overlapping 1 Mbp windows

In the case of Ct values from RT-qPCR the conducted GWAS did not detect any statistically significant SNPs for the Bonferroni criterion (Fig. 6a; α = 0.05). In terms of explained additive genetic variance a region accounting for ~ 2.4% was detected on chromosome four (Fig. 6b).

### Prediction of BKD-resistant charr

At first glance, the use of genomic prediction appeared promising for selecting BKD-resistant charr. For surviving days, the accuracy, in terms of Pearson correlation, between the predicted DGVs of the validation sets and their corresponding phenotypes ranged from 0.41 to 0.52 (Table 2). The mean accuracy was 0.46, with an accompanying standard error of 0.01. A relatively small over- and deflation was observed, with the regression slope coefficient metric ranging between 0.87 and 1.11 (Table 2).

**Table 2:** Prediction accuracies and inflations of GBLUP derived DGVs for surviving days post infection

3-fold CV	Replicate 1	Replicate 2	Replicate 3	Replicate 4	Replicate 5
Prediction accuracies					
1st fold	0.48	0.47	0.45	0.45	0.48
2nd fold	0.50	0.42	0.46	0.52	0.47
3rd fold	0.43	0.44	0.45	0.49	0.45
**Inflations**					
1st fold	1.02	0.98	0.97	0.89	1.03
2nd fold	1.11	0.87	0.95	1.10	0.99
3rd fold	0.89	0.90	0.94	1.07	0.94

The accuracies represent Pearson correlation coefficients between direct genomic values (DGVs) and the number of surviving days. The inflation metrics were derived from regressing the adjusted for fixed-effects phenotypes on the DGVs. Values above one suggests deflated DGVs, while values below one suggest inflated DGVs. A 3-fold CV (cross-validation) strategy was performed, replicated 5 times

A large drop in prediction accuracy was observed when the same cross-validation strategy was applied to Ct values from RT-qPCR. Accuracies ranged between 0.02 and 0.13 (Table 3). At the same time, the DGVs were substantially over- or deflated with the values of the regression slope coefficient ranging between 0.24 and 2.53 (Table 3).

**Table 3:** Prediction accuracies and inflations of GBLUP derived DGVs for RT-qPCR Ct values

3-fold CV	Replicate 1	Replicate 2	Replicate 3	Replicate 4	Replicate 5
Prediction accuracies					
1st fold	0.09	0.07	0.02	0.07	0.02
2nd fold	0.06	0.14	0.05	0.03	0.06
3rd fold	0.05	0.05	0.13	0.07	0.05
**Inflations**					
1st fold	1.24	1.02	0.24	0.95	0.30
2nd fold	0.80	2.53	0.85	0.45	0.81
3rd fold	0.90	0.60	2.32	1.20	0.66

The accuracies represent Pearson correlation coefficients between direct genomic values (DGVs) and the Ct values from RT-qPCR. The inflation metrics were derived from regressing the adjusted for fixed-effects phenotypes on the DGVs. Values above one suggest deflated DGVs, while values below one suggest inflated DGVs. A 3-fold CV (cross-validation) strategy was performed, replicated 5 times

**Fig. 7:**
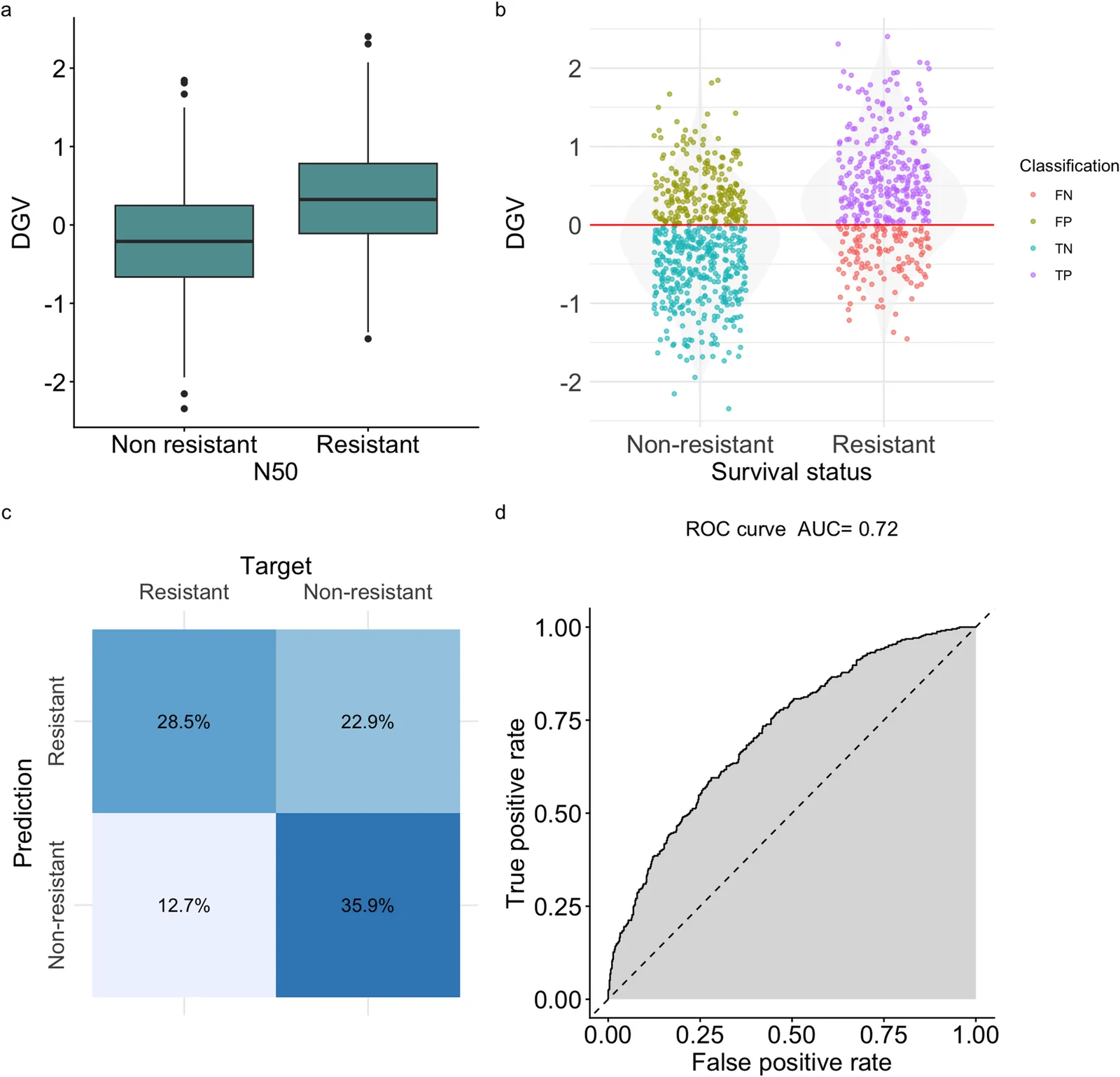
BKD resistance prediction analysis based on N_50_ survival. (**a**) Boxplot comparing the distribution of direct genomic values (DGV) between non-resistant and resistant groups. (**b**) A scatterplot depicting the classification categories in different colours versus the masked phenotype. (**c**) Confusion matrix displaying the classification performance of the prediction model, showing percentages for True Positives (TP; 28.5%), False Positives (FP; 22.9%), False Negatives (FN; 12.7%), and True Negatives (TN; 35.9%). (**d**) ROC curve and corresponding AUC metric

For N_50_ survival, prediction efficiency was assessed by first inspecting boxplots showing the range of DGVs among resistant and non-resistant charr in the test set (animals from each of the two tanks of the challenge experiment were, in turn, used as the test set) (Fig. 7a). In addition, the classification outputs were distinguished into true positives, true negatives, false positives, and false negatives (Fig. 7b). Thereafter, classification was assessed more formally using a confusion matrix (Fig. 7c). The correct classifications (the sum of true positives and true negatives) accounted for 64.4%. Regarding classification errors, false negatives accounted for 12.7% of predictions, while false positives accounted for 22.9%. As an overall metric for assessing classification performance, we used the AUC from the ROC curve. The AUC was 0.72 (Fig. 7d). Finally, no obvious differences were found in any of the aforementioned metrics when either tank was used as the test set.

### Associations between growth and BKD EBVs

**Fig. 8:**
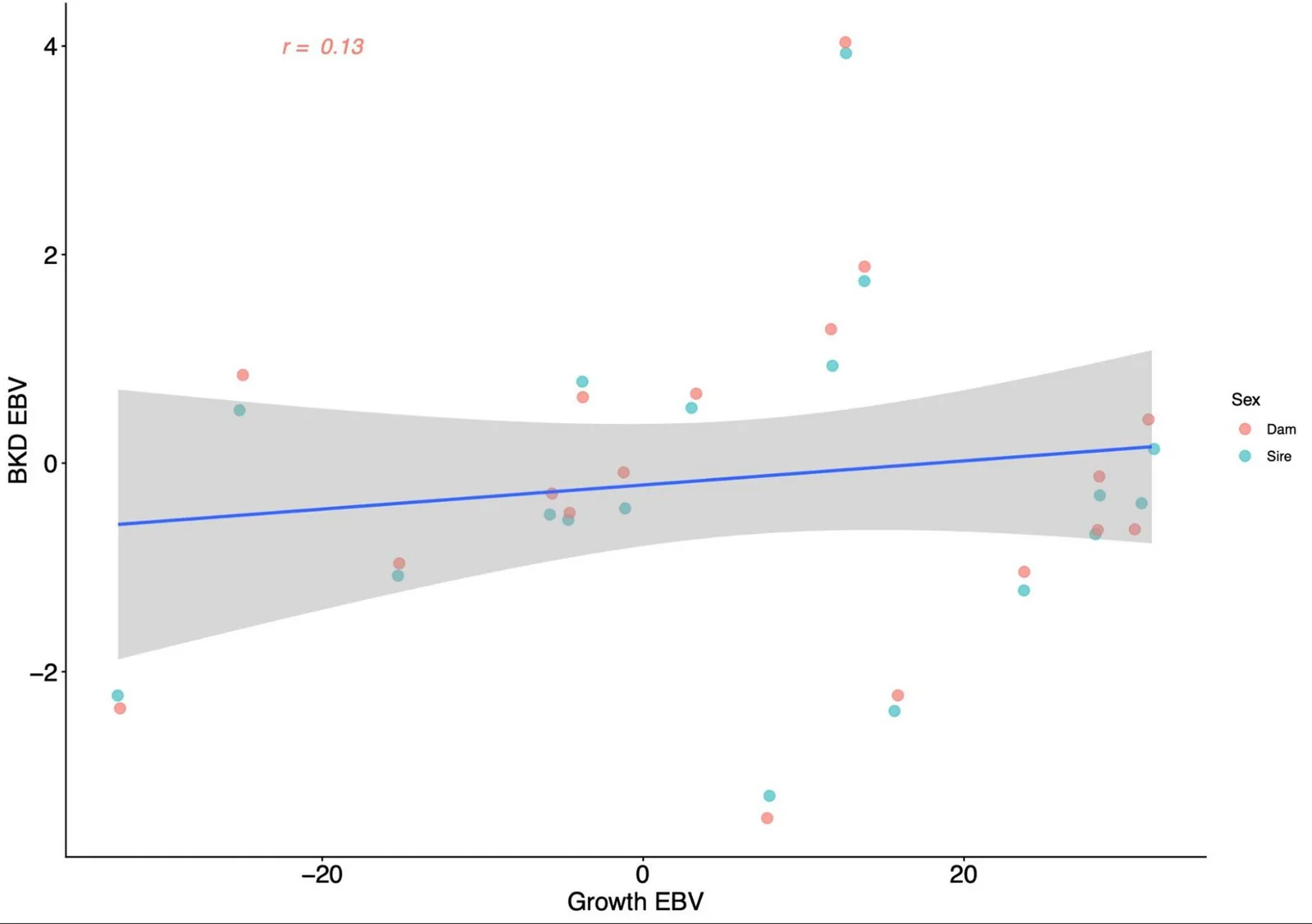
Relationship between BKD resistance and growth estimated breeding values (EBVs). The blue line represents the linear regression fit, with the grey shaded region indicating the 95% confidence interval. Individual data points represent the EBVs of single animals, colour-coded by sex (sires vs. dams)

Only a weak, non-significant positive trend was observed between BKD resistance (measured in terms of surviving days) and growth EBVs. Specifically, regressing BKD resistance on growth EBVs yielded a regression coefficient of 0.012 (SE = 0.015; *P* = 0.43), while the Pearson correlation coefficient was 0.13 (Fig. 8).

## Discussion

A medium-density SNP array (71k) for Arctic charr was developed and used to assess the potential to select for BKD-resistant animals. As previously mentioned, the new array essentially contains a subset of the most informative and reproducible SNPs from a recently developed high-density array (600,000 SNPs) in the same population [27]. Genomic selection practices in animal breeding typically rely on medium-density SNP arrays (~ 57k) as a more economically viable option. Densities of this magnitude also align with the relatively low N_e_ that characterises many aquaculture breeding programs [55].

The newly developed array appears well-suited to the Swedish Arctic charr breeding program. Among other things, it circumvents one of the most highlighted weaknesses of SNP arrays, namely ascertainment bias when an array is developed in one population and used in others [56]. Critically, only a minimal number of SNPs were discarded (~ 2.8%), even though strict thresholds were implemented using quality control (e.g., minimum call rate of 98%). For the size of the Arctic charr breeding program, a large number of animals, encompassing practically the entire number of families in the latest year class, were genotyped (~ 2000). This high number of retained SNPs suggests that this array could play a pivotal role in the coming years by providing high-quality genomic data. In comparison, it is typical in salmonid breeding studies to discard 10% or more of the array’s SNPs, even with less stringent filtering thresholds [14, 57] or even when applied to the same population from which the array was initially produced [58]. Of course, to be more objective, it would be fair to claim that the newly developed array has a relatively narrow scope of applicability. Nevertheless, because the high-density array [27] from which the current array originates included information from other Nordic Arctic charr populations, its usefulness might not be restricted to the Swedish population only.

Our newly developed arr​ay was used here to assess the potential to select for BKD-resistant charr. An initial interpretation of the challenge trial results suggests that Arctic charr are particularly vulnerable to *Renibacterium salmoninarum*, with only approximately 2% of the challenged population surviving the six-week observation period. As no prior information was available on how pathogenic the chosen strain would be for the Swedish Arctic charr, a relatively high concentration was chosen to ensure that disease symptoms, and at least some mortality, would be induced during the trial. For the same reason, we chose IP injection over a model with more natural bacterial transmission from infected fish through cohabitation. The latter approach would also have made the trial time much longer and more expensive. Furthermore, cohabitation challenges carry inherent limitations as well, as uniform pathogen exposure across all tested individuals cannot be guaranteed.

Regardless this high mortality rate appears to be in line with previously documented acute outbreaks in the species, where mortality reached up to 80% [4]. Since a controlled experimental setup was used here and the fish were injected with a bacterial inoculum, this discrepancy does not seem far-fetched. In contrast, previous studies in Atlantic salmon that also used an IP infection method reported lower mortalities ranging from 56% to 65% [26, 59]. Those results suggest that Arctic charr might be more susceptible to BKD than Atlantic salmon. This also agrees with the fact that the bacterial cells injected here (5 × 10^6^ CFU/fish) were orders of magnitude lower than those previously used in Atlantic salmon (10^8^ CFU/fish) [26]. However, as the fish of the aforementioned studies were substantially larger (40 g and over 500 g), such generalization might not be fully accurate. At the same time, it is possible that the bacterial strain in our study was more virulent than those previously used in Atlantic salmon. Regardless, a natural question arises: how realistic is our experimental setting? Obviously, an IP injection does not reflect the actual route of infection, as it immediately exposes the fish to an unnaturally high infectious dose that bypasses the host’s primary defense mechanisms. In contrast to the acute disease seen in this challenge, naturally infected fish often become chronically infected with a slowly progressive disease. The immune system tries to kill the bacteria and limit their spread by forming granulomas, but *R. salmoninarum* can escape by hiding inside white blood cells that phagocytose them. This characteristic makes antibiotic treatment and vaccine extremely challenging [1]. Although a conclusive answer towards the extent to which the results of the current study are transferable to the field is not possible, it is worth mentioning that aquaculture breeding for disease resistance has commonly relied on challenge tests using IP injections to infect the population under study, even in commercial programs [7, 60, 61].

Several phenotypic proxies derived from the BKD challenge experiment were used in our study to investigate the underlying genetics, beginning with the estimation of relevant variance components. A motivation for using different proxies also stems from prior observations in aquaculture breeding studies on disease resistance, suggesting that overall survival on its own might be an inadequate measure of resistance [62]. More specifically, the proxies used were the number of surviving days both as a continuous and a right–censored trait and the overall and N_50_ survival as binary traits. In terms of N_50_ it is worth mentioning that it has been routinely used in disease challenge experiments in Atlantic salmon where the obtained variance components and the predictive ability of the animal models were similar, though lower, to those where final survival was used [63]. Overall, moderate heritability estimates were obtained (0.16–0.56), suggesting there is potential for applying selective breeding practices. This range of heritability values is consistent with values previously documented for various diseases in salmonids [64–66]. It should be noted, though, that especially in the case of overall survival, the heritability estimate (0.26) was accompanied by a substantial standard error (0.15). Furthermore, the Gibbs sampler exhibited poor mixing and high autocorrelation, indicating that the posterior was sampled inefficiently and the resulting estimate is likely unreliable. This inefficiency stems from the high degree of skewness in the data, a condition under which the Gibbs sampler often fails to explore the posterior distribution effectively [67]. As an alternative, we performed the same analysis using a Hamiltonian Monte Carlo based sampler called NUTS. Such algorithms have been shown to be promising alternatives to Gibbs sampling, in animal breeding as well, due to their better sampling attributes, which allow for a more efficient search of the posterior distribution [68]. However, in our case, the obtained posterior density intervals remained wide, suggesting a data limitation in accurately estimating the variance component in question rather than an algorithmic one.

In contrast to the moderate heritability estimates that were observed for surviving days and overall or N_50_ survival, a low heritability (0.054) was found for Ct values from RT-qPCR. Nevertheless, the latter estimate was based on only 800 records compared to 1982 for the other traits. Furthermore, because our sampled subset was not random, potential biases exist. In addition, it could well be that all those traits capture different aspects of disease resilience, which may differ in magnitude, thereby explaining the large differences between the heritability estimates. In theory, disease resilience refers to the host’s ability to maintain performance levels close to those of healthy animals while infected and can be decomposed to disease resistance and tolerance that refer to the host’s ability to limit the pathogen load or the damage caused by the pathogen, respectively [36, 69]. Notably, the underlying mechanisms amongst them have been suggested to differ [34]. Critically, in our study, it does not seem possible to disentangle those factors partly because of the high mortality we observed. Aside that a more elaborate experimental design would be required [70]. Nevertheless, despite the aforementioned limitations, valuable information was extracted. The relationship between survival and bacterial load, quantified through Ct values from RT-qPCR, yielded a weak negative Pearson correlation of -0.15. While lower Ct values indicate higher bacterial loads, the weak nature of this correlation suggests that the genetic mechanisms governing survival time may be partially distinct from those governing pathogen proliferation. This distinction is critical for breeding programs; selecting solely for survival may not necessarily result in resistance (the ability to limit bacterial load), and vice versa. In that aspect, breeding for disease resilience might be a more effective strategy [36]. Furthermore, a strategy combining genetic and epidemiological models could be more optimal [35, 71]. The main obstacle though, in the above, is the design of a cost-effective approach for relevant phenotypes which especially for aquaculture species is particularly challenging [7].

The GWAS (Fig. 5) provided the first insights into the genetic architecture of BKD resistance in Arctic charr, suggesting that BKD resistance is polygenic. It is worth noting that, with few exceptions, most aquaculture breeding studies reported polygenic inheritance in disease resistance [7, 15], favoring the application of genomic prediction over marker-assisted selection. The ability to accurately predict resistant individuals (e.g. AUC = 0.72) using the medium-density array demonstrates that genomic selection is a viable tool for improving BKD resistance in Arctic charr. Incorporating these genomic breeding values into the national program, which currently relies on pedigree-derived breeding values, will enable the selection of resistant candidates lacking phenotypic data, thereby significantly increasing selection accuracy. Nevertheless, validation with data from field outbreaks will be necessary beforehand. Furthermore, a pending issue that would still need to be addressed is the possibility that such candidates can still infect their offspring, as BKD is transmitted vertically as well.

In light of the above, it is important to stress that before adjusting the overall breeding goals, it is pivotal to ensure that no unfavorable associations exist among the selected traits. In our particular case, the focus would be on ensuring that selecting for increased BKD resistance would not negatively affect selection for increased growth. Unfortunately, because only a subset of the parental generation was genotyped, we performed only a preliminary analysis (involving 36 broodfish). Despite the fact that no conclusive inferences can be made, it appeared promising that only a slight and positive trend was observed amongst the breeding values of those two traits. This suggests that selecting for BKD resistance should not negatively affect selection for increased growth. However, to form a more complete picture, additional genotyping would be required.

## Supplementary Information

Below is the link to the electronic supplementary material.

Supplementary Material 1: Additional file 1 Table S1. File Format: docx. Title: Estimated variance components for continuous traits related to BKD resistance in Arctic charr. Additional file 1 Table S2. File Format: docx. Title: Estimated variance components for binary traits related to BKD resistance in Arctic charr

Supplementary Material 2: Additional file 2 Figure S1. File Format: docx. Title: Family size distribution. Additional file 2 Figure S2. File Format: docx. Title: GWAS for surviving days

## Data Availability

The code of the conducted analysis is available through https://github.com/chpalaiokostas/Genetic_resistance_BKD. The SNP genotypes are available upon reasonable request.
