# Supplementary material for: Genomic insights into bacterial kidney disease resistance in Arctic charr (Salvelinus alpinus) using a newly developed 71k SNP array
Source: Genet Sel Evol. 2026 Nov 14;58:92. doi: 10.1186/s12711-026-01118-w (PMC13769156; doi:10.1186/s12711-026-01118-w)
Supplement: Supplementary file 1 [file 12711_2026_1118_MOESM1_ESM.docx]

**Table S1.** Estimated variance components for continuous traits related to BKD resistance in Arctic charr

| **Trait** | **Additive genetic variance (SE)** | **Residual variance (SE)** |
| --- | --- | --- |
| Surviving days | 7.45 (0.88) | 8.16 (0.39) |
| Ct (RT-qPCR) | 0.23 (0.16) | 4.3 (0.25) |

SE denotes standard error.

**Table S2.** Estimated variance components for binary traits related to BKD resistance in Arctic charr

|  | **Posterior Distribution Mean** | **Highest Posterior Density (95% interval)** | **Autocorrelation – lag 1** |
| --- | --- | --- | --- |
| **N50 survival** | | | |
| Additive genetic variance | 1.3 | 0.86 – 1.75 | 0.032 |
| Residual variance | 1 | 0.91 – 1.09 | 0.012 |
| **Overall survival** | | | |
| Additive genetic variance | 0.45 | 0.64e-03 – 1.22 | 0.392 |
| Residual variance | 1 | 0.92 – 1.09 | -0.011 |
