# Supplementary material for: Genomic insights into bacterial kidney disease resistance in Arctic charr (Salvelinus alpinus) using a newly developed 71k SNP array
Source: Genet Sel Evol. 2026 Nov 14;58:92. doi: 10.1186/s12711-026-01118-w (PMC13769156; doi:10.1186/s12711-026-01118-w)
Supplement: Supplementary file 2 [file 12711_2026_1118_MOESM2_ESM.docx]

**Figure S1.** Family size distribution

**Figure S2.** Manhattan plot depicting -log_10_(p-values) estimated from WGBLUP for surviving days across the Arctic charr genome. Each SNP is depicted by a separate dot of alternating colours across the different chromosomes.
